# Cognitive Impairment in Type 2 Diabetes Mellitus

**DOI:** 10.7759/cureus.22193

**Published:** 2022-02-14

**Authors:** Aimen Malik, Mubariz Ahmed, Sarah Mansoor, Saima Ambreen, Basil Usman, Malik Shehryar

**Affiliations:** 1 Emergency Medicine, Holy Family Hospital, Rawalpindi, PAK; 2 Internal Medicine, Holy Family Hospital, Rawalpindi, PAK; 3 Internal Medicine, Diana, Princess of Wales Hospital, Grimsby, GBR; 4 Medical School, Rawalpindi Medical University, Rawalpindi, PAK

**Keywords:** cognitive, brain, diabetes, encephalopathy, mmse

## Abstract

Objective

To determine the incidence of cognitive impairment established on the mini-mental state assessment in type 2 diabetic patients presenting at Holy Family Hospital, Rawalpindi.

Materials and methods

This cross-sectional descriptive study was carried out from June 2019 to December 2019. Individuals with a diagnosis of type 2 diabetes mellitus were included, and detailed history, physical examination, and biochemical variables were noted. They were assessed through Mini-Mental State Examination (MMSE) (Urdu translation) to look for the primary outcome variable, i.e., cognitive impairment. All patients with type 2 diabetes mellitus diagnosed at least one year back, irrespective of gender, were included in this investigation. Patients with a previous history of head injury, epilepsy, stroke, those on an antidepressant or antipsychotic medications, those with deranged renal function tests, and those already diagnosed with dementia were excluded from the study.

Results

Three hundred thirty-two patients meeting the inclusion criteria were included in the study. The mean ± standard deviation age of the study population was 65.32 ± 11.33 years, with maximum age being 80 years and the minimum being 50 years. Two hundred patients (60.24%) were below 65 years of age, and 132 patients (39.76%) were 65 years of age or above. Two hundred sixteen (65.06%) were males, and 116 (34.96%) were females. The mean duration of diabetes mellitus (DM) was 10.17 ± 4.81. The mean MMSE score was 22.69 ± 5.26. Out of 332 patients, 81 (24.4%) patients had cognitive impairment. Patients who were 65 or older had a significantly higher proportion of cognitive impairment, compared to those below 65 years of age (p-value = 0.0214). There was no significant difference in the proportion of cognitively impaired patients between males and females (p-value = 0.2497). Similarly, there was no significant difference between those who were diagnosed with type 2 diabetes for 10 years or more and those who were diagnosed less than 10 years ago (p-value = 0.3791).

Conclusion

Cognitive impairment is common in individuals having type 2 diabetes mellitus. It is also associated with the increasing age of diabetic patients. However, cognitive impairment in type 2 diabetes mellitus is not associated with gender. In addition, there is no significant difference in cognitive impairment between the patients who were diagnosed with diabetes more than 10 years ago and those who had it diagnosed less than 10 years ago.

## Introduction

Diabetes mellitus (DM) is a chronic disorder of elevated blood glucose. It is classified into two distinct types (type 1 and 2). Type 1 diabetes mellitus is characterized by autoimmune destruction of beta cells in the pancreas leading to absolute insulin deficiency. Type 2 diabetes mellitus is characterized by different combinations of insulin resistance and insulin deficiency. As per WHO, diabetes is diagnosed with one or a combination of the following criteria: a fasting plasma glucose concentration equivalent to or greater than 7.0 mmol/liter (126 mg/dL), two hours plasma glucose concentration equivalent to or greater than 11.1 mmol/liter (200 mg/dL) during a 75-g oral glucose tolerance test, and/or hemoglobin A1C (HbA1c) equivalent to or beyond 6.5% (48 mmol/mol) [1].

Diabetes occurs in 5%-10% of the population. Majority of the affected population (95%) has type 2 diabetes mellitus. It is estimated 463 million people were affected with diabetes in 2019 [2]. Over the past 25 years, the prevalence of diabetes has doubled in men and increased by 60% in women [3]. In Pakistan, the overall weighted prevalence of diabetes was recorded up to 26.3% [4].

Various micro- and macrovascular complications of diabetes mean that the disease is a major factor of morbidity and mortality worldwide. These include cardiovascular and cerebrovascular disease, peripheral vascular disease, retinopathy, and end-stage renal disease [5]. Diabetes multiplies the risk of all-site cancer (except prostatic carcinoma) [5]. It also increases the risk of mental health illnesses among patients, such as depression [6]. These complications are generally well known to the healthcare providers and are usually dealt with adequately, according to the resources available.

However, a problem commonly ignored by healthcare providers is the link between diabetes and cognitive dysfunction. This is despite the relationship being well established in the literature. In recent years, several studies have shown strong evidence for the presence of cognitive impairment in diabetes [7-9]. A recent systematic review and meta-analysis estimated the prevalence of mild cognitive impairment type 2 diabetes mellitus (T2DM) patients to be as high as 45% [10].

Insulin resistance and obesity (which are shared risk factors for both diabetes and cognitive impairment), chronic low-grade inflammation and hyperglycemia or hypoglycemia, and cardiovascular complications of diabetes may be some of the risk factors implicated in development of cognitive impairment in patients [11,12]. Diabetes is known to adversely affect several cognitive domains. These include memory and processing speed, although executive function may be spared [13,14].

Since there is a dearth of studies conducted in our region exploring the relationship between type 2 diabetes and cognitive impairment, we wanted to conduct this investigation. Knowledge of this prevalence would help healthcare providers give it due consideration while managing the complications of diabetes. 

## Materials and methods

This descriptive cross-sectional study was conducted at Medicine Unit I, Holy Family Hospital, Rawalpindi, Pakistan, from June 2019 to December 2019. Ethical approval was taken from the institutional research forum of the Rawalpindi Medical University. Patients with age greater than 50 years, random blood sugar greater than 200 mg/dL, fasting blood sugar level greater than 126 mg/dL, or HbA1c levels greater than 6.5% were defined as having type 2 diabetes mellitus. All patients with type 2 diabetes mellitus diagnosed at least one year back, irrespective of gender, were included in the study. Patients with a history of head injury, epilepsy, stroke, those on an antidepressant or antipsychotic medication, those with deranged renal function tests, and those already diagnosed with dementia were excluded from the study. Three hundred thirty-two patients were selected using consecutive (nonprobability) sampling. All the patients were explained about the nature and purpose of the study. Informed written consent for participation was obtained. Patients’ sociodemographic details including years of education were recorded in a proforma.

An Urdu translation of Mini-Mental State Examination (MMSE) was applied through the interview from each patient to assess cognitive impairment. The MMSE takes into account various domains of cognitive functioning. These include spatial and temporal orientation, immediate memory, attention/concentration, delayed recall, and language [15]. Patients with scores 23 or below out of 30 were considered cognitively impaired. The authors, without prior knowledge that the tool had been licensed, used unauthorized copies of Urdu MMSE for data collection. Permission for publication of data obtained from administering MMSE to 332 patients was retroactively obtained from Psychological Assessment Resources, Inc.

The collected data were analyzed using the statistical software SPSS-17 (IBM, New York, United States). For categorical variables like gender and presence or absence of cognitive impairments, frequencies along with percentages were calculated. For quantitative variables like age and the exact MMSE score, the mean and standard deviation was calculated. Factors such as age, gender, and duration of diabetes were controlled through stratification. Chi-square test was applied poststratification. P-value less than 0.05 was considered significant.

## Results

**Figure 1:**
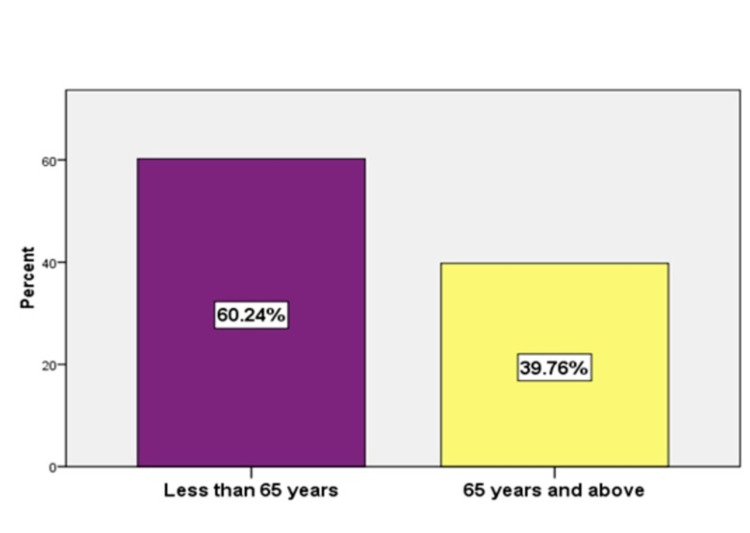
Distribution of patients by age

**Figure 2:**
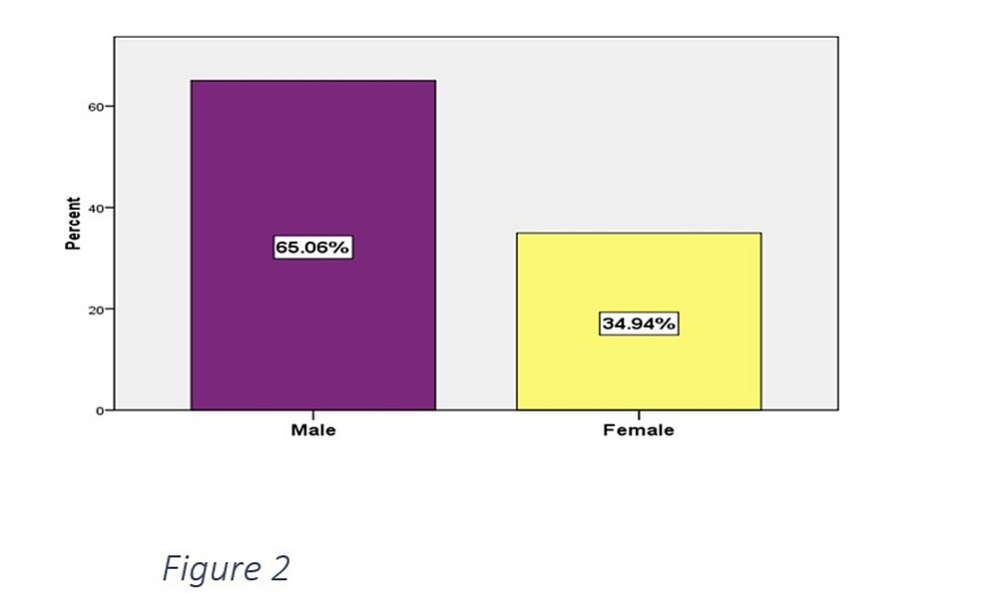
Distribution of patients by gender

The mean ± standard deviation age of the study population was 65.32 ± 11.33 years, with the maximum age being 80 years and the minimum being 50 years. Two hundred patients (60.24%) were below 65 years of age, and 132 patients (39.76%) were 65 years of age or above (Figure 1). Two hundred sixteen (65.06%) were males, and 116 (34.96%) were females (Figure 2). The mean duration of DM was 10.17 ± 4.81. The mean MMSE score was 22.69 ± 5.26.

**Figure 3:**
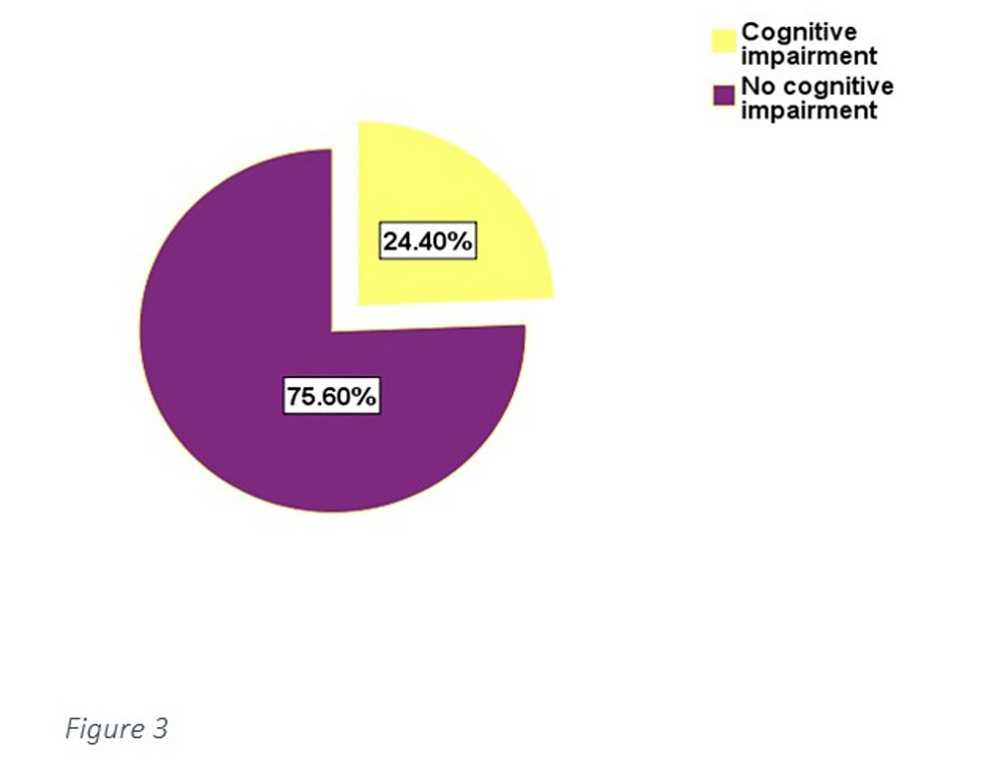
Proportion of cognitively impaired patients versus those not cognitively impaired

Out of 332 patients, 81 (24.4%) patients had cognitive impairment (Figure 3). Patients who were 65 or older had a significantly higher proportion of cognitive impairment, compared to those below 65 years of age (p-value = 0.0214) (Table 1). There was no significant difference in the proportion of cognitively impaired patients between males and females (p-value = 0.2497) (Table 1). Similarly, there was no significant difference in cognitive impairment between those who had type 2 diabetes mellitus for 10 years or more and those who had type 2 diabetes mellitus for less than 10 years (p-value = 0.3791) (Table 1).

**Table 1:** Analysis of cognitive impairment with age, gender, and duration of type 2 diabetes mellitus T2DM: type 2 diabetes mellitus.

Age						
	Less than 65 years (total 200)		More than 65 years (total 132)	p-value		
Patients with cognitive impairment	40 (20%)		41 (31.1%)	0.0214		
Gender						
		Males (total 216)	Females (total 116)		p-value	
Patients with cognitive impairment		57 (26.4%)	24 (20.7%)		0.2497	
Duration of T2DM						
		More than 10 years (total 102)	Less than 10 years (total 230)			p-value
Patients with cognitive impairment		28 (27.5%)	53 (23.0%)			0.3791

## Discussion

The results of our study ascertain that almost one in four of the patients suffering from type 2 diabetes mellitus also suffers from cognitive impairment; therefore, cognitive dysfunction is not uncommon in patients with T2DM. This proportion is in line with the findings in the literature. Khullar et al. found this proportion to be 33.73% [16]. Other studies have shown it to range from 3% to 23% [17,18].

We want to emphasize that cognitive dysfunction in type 2 diabetes mellitus is almost as common as its other micro- and macrovascular complications. Awareness of this important complication can help in better counseling of the patients and their families. It can lead to directing more material resources and expertise toward the issue. It can also lead to a better multidisciplinary approach, such as the involvement of the neurologist for better patient management.

Cognitive impairment might be one of the multitudes of reasons for poor compliance of the patients to lifestyle modifications and pharmacotherapy used to manage the disease. Patient education regarding cognitive impairment would help them better understand the problems they are facing. This might lead to better compliance to treatment and, as a result, good glycemic control. Also, education of the family and other caregivers regarding the presence of cognitive dysfunction might help them better understand the problems the patient is facing, and as a result, patient care would improve. In summary, awareness of cognitive dysfunction in type 2 diabetics among physicians, patients, and their caregivers can go a long way in improving patients’ quality of life.

In addition to this, our results show that increasing age is a risk factor for the development of cognitive impairment in type 2 diabetics. This relationship is also well established in the literature [19]. Since diabetes mellitus [20] and old age are both independent risk factors for developing Alzheimer’s disease, vascular dementia, and other disorders resulting in the culmination of cognitive decline, a combination of both can contribute to a higher incidence of cognitive impairment among older diabetics.

The present study showed no significant difference in cognitive impairment between the genders. This is in contrast to other studies, which have described that the diabetic women have double the risk of neurocognitive impairment compared to men, and female gender is an independent risk factor for development of neurocognitive deficit [21,22]. One study even described a 3.75 times risk of cognitive impairment in women compared to men [23]. Perhaps, a larger sample size could have shed more light on this relationship.

The present study also showed no significant relationship between the duration of T2DM with cognitive impairment. This is also contrary to many previous studies investigating the association. One study showed that patients who have been diagnosed with T2DM for five years or more perform worse in aspects of cognition such as logical memory and word fluency compared to those who have been diagnosed newly [24]. A study of the Iranian population reported a negative correlation between MMSE scores and the duration of T2DM [25]. Similarly, a study conducted in India showed that diabetic individuals with a duration of diabetes for more than 10 years were 4.34 times more likely to develop cognitive impairment compared to the newly diagnosed diabetics [26]. We propose that more studies should be conducted in our region, so this relationship could be explored further.

Our results might have been influenced by certain potential limitations. Firstly, age and education can influence MMSE scoring to judge neurocognition. Secondly, since the sensitivity of MMSE for timed elements of executive functions and memory is low, residual confounding for them may have remained to be exposed. Thirdly, the number of diabetic patients included in the study might be small and a larger cohort could have better elucidated the relationships we investigated.

## Conclusions

Cognitive impairment is common in individuals having type 2 diabetes mellitus. It is also associated with the increasing age of diabetic patients. However, cognitive impairment in type 2 diabetes mellitus is not associated with gender. In addition, there is no significant difference in cognitive impairment between the patients who were diagnosed with diabetes more than 10 years ago and those who had it diagnosed less than 10 years ago.
