# Correction: Cognitive Impairment in Type 2 Diabetes Mellitus

**DOI:** 10.7759/cureus.c59

**Published:** 2022-03-18

**Authors:** Aimen Malik, Mubariz Ahmed, Sarah Mansoor, Saima Ambreen, Basil Usman, Malik Shehryar

**Affiliations:** 1 Emergency Medicine, Holy Family Hospital, Rawalpindi, PAK; 2 Internal Medicine, Holy Family Hospital, Rawalpindi, PAK; 3 Internal Medicine, Diana, Princess of Wales Hospital, Grimsby, GBR; 4 Medical School, Rawalpindi Medical University, Rawalpindi, PAK

This article has been corrected to resolve an issue regarding attribution of the licensed tool used in data collection. The authors started data collection in June 2019 with an Urdu translation of Mini Mental State Examination (MMSE) that was commonly used and in circulation in Pakistan for judging cognitive status of patients. Unfortunately at that time the authors did not know that the MMSE had been licensed, and that they would have to purchase authorized copies from the copyright holder Psychological Assessment Resources, Inc for use in their study. This issue was brought to their attention prior to publication and the authors then contacted the license holding company for retroactive permission to publish and paid for the rights.

Per the permission agreement, the authors were required to state in the publication that unauthorized Urdu MMSE copies were used. This was added to the Intellectual Property disclosures section with the authors believing they had satisfied this condition. However, after publication, Psychological Assessment Resources required the following corrections, which have since been made:

- The following statement was moved from the Disclosures section to the second paragraph of the Materials and Methods section: "The authors without prior knowledge that the tool had been licensed used unauthorized copies of Urdu MMSE for data collection. Permission for publication of data obtained from administering MMSE to 332 patients was retroactively obtained from Psychological Assessment Resources, Inc."
- Since the copies of Urdu MMSE were unauthorized, they cannot be validated and the reference to validation should be removed. As a result, the following sentence and accompanying reference have been removed and the remaining in-text citations renumbered to ensure accuracy with the revised reference list: "The Urdu translation of MMSE has previously been validated, and a cut-off value of 24 was found to maximize sensitivity and specificity (69% and 93%, respectively) when subjects of all education levels were collectively considered [16]." [16] Awan S, Shahbaz N, Akhtar SW, et al.: Validation study of the Mini-Mental State Examination in Urdu language for Pakistani population. Open Neurol J. 2015, 9:53-8. 10.2174/1874205X01509010053

